# Analysis of complement biomarkers in systemic sclerosis indicates a distinct pattern in scleroderma renal crisis

**DOI:** 10.1186/s13075-016-1168-x

**Published:** 2016-11-18

**Authors:** Marcin Okrój, Martin Johansson, Tore Saxne, Anna M. Blom, Roger Hesselstrand

**Affiliations:** 1 Department of Translational Medicine, Section of Medical Protein Chemistry, Lund University, Inga Marie Nilssons Street 53, Malmö, S-20502 Sweden; 2 Department of Medical Biotechnology, Intercollegiate Faculty of Biotechnology UG-MUG, Medical University of Gdańsk, Gdańsk, 80210 Poland; 3 Department of Translational Medicine, Section of Clinical Pathology, Lund University, Jan Waldenströms street 59, Malmö, S-20502 Sweden; 4 Department of Clinical Sciences, Lund, Section of Rheumatology, Lund University, Skåne University Hospital, Lund, S-22185 Sweden

**Keywords:** Scleroderma, Complement, Biomarkers, Renal crisis, Systemic sclerosis

## Abstract

**Background:**

The complement system has been implicated in pathogenesis of systemic sclerosis (SSc). The goal of the present study was to evaluate improved complement biomarkers in SSc.

**Methods:**

The presence of C4d, reflecting activation of the classical/lectin pathways, C3bBbP corresponding to activation of the alternative pathway, and soluble terminal complement complexes (all complement pathways), was measured in plasma samples by enzyme-linked immunosorbent assay and correlated to clinical parameters. The study included 81 patients with limited cutaneous SSc and 41 with diffuse cutaneous SSc, as well as 47 matched healthy controls and 81 patients with rheumatoid arthritis, 22 with psoriatic arthritis and 20 with ankylosing spondylitis. Skin and kidney biopsies of selected patients were stained to detect deposited C3b as a marker of local complement activation.

**Results:**

Biomarkers of activation of all complement pathways were increased in SSc compared with healthy controls and were similar to those in other rheumatic diseases. When patients with SSc were divided into subgroups, a distinct pattern of complement markers was observed in individuals with scleroderma renal crisis (SRC). By functional assay, we confirmed a significant decrease in complement haemolytic activity in SRC vs. non-SRC patients, indicating complement consumption. Further, we detected glomerular deposits of C3b in some patients with SRC.

**Conclusions:**

The data indicate that complement activation is an important feature of SRC.

## Background

Scleroderma, also termed *systemic sclerosis* (SSc), is an autoimmune disease of connective tissue. Its pathology involves excessive collagen production, resulting in fibrosis of skin and internal organs [1, 2]. This condition is accompanied by microangiopathy of varying severity and locations, most obviously seen as Raynaud’s phenomenon. The most widely accepted classification distinguishes two main subtypes: limited cutaneous SSc (lcSSc) and diffuse cutaneous SSc (dcSSc) [3]. In the latter case, internal organs, most typically the kidneys, gastrointestinal tract, heart and lungs, are more severely affected. There is an ongoing discussion about the primary cause of SSc because many molecular patterns and various pathways have been found to be involved in the pathogenesis. Importantly, 90% of patients with SSc present with autoantibodies to intracellular components such as topoisomerase, centromeres, histones, RNA polymerases or ribonucleases, and these patients also show an increase in surface density of CD19 on their B cells [2]. It has been shown that the presence of these autoantibodies represents specific phenotypes of the disease, but less is known about their pathogenic role.

Data from in vivo models show that low expression of CD19 affects B-cell proliferation, whereas overexpression potentiates antibody production and increases the degree of autoantibodies [4]. Indeed, whole-genome microarray analysis has demonstrated that gene expression patterns characteristic of plasma cells decreases more than 90% upon anti-CD19 treatment and correlates with inhibition of collagen expression [5]. Apart from intracellular components, protein complexes present on the surface of fibroblasts, lymphocytes and endothelial cells are also targets of autoantibodies in SSc [6]. These autoantibodies may activate fibroblasts to produce collagen, either directly or indirectly, by fuelling local inflammation and release of pro-inflammatory cytokines. However, it is unclear to what extent the complement system, for which antibodies are a main trigger, contributes to SSc pathogenesis. CD21 (CR2), a receptor on the surface of B cells, binds activation products of the main complement factor C3b. Following complement activation, C3b covalently binds target surfaces and forms transient enzymatic complexes: complement convertases such as C3bBbP, which fuel downstream events of the cascade such as release of the potent pro-inflammatory anaphylatoxin C5a, and formation of terminal complement complexes (TCCs), which can cause cell lysis. CD21 and CD19 associate and form a signal transduction complex capable of enhancing B-cell responses to antigen once CD21 binds complement degradation fragments [7].

In fact, over the last 30 years, researchers have tried to correlate the levels of complement proteins, markers of complement activation and circulating immune complexes in patients’ bloodstream with severity of SSc and different subtypes of the disease. Elevated immune complexes were found only in some patients and were not associated with clinical or serological features [8, 9]. In another study, low-molecular-weight markers of complement activation—Ba, C3d and C4d—were measured by nephelometry in plasma of patients with SSc [10]. The results showed that C3d, C4d and Ba fragments, as well as C3d:C3 and C4d:C4 ratios, were clearly higher in patients with SSc than in healthy control subjects, indicating increased complement activation. Also, patients with dcSSc showed significantly higher values than those with lcSSc [10]. On the basis of observations of higher C4d values in patients with SSc and subendothelial deposition of immune complexes [11], the classical complement pathway may indeed play a role in the pathogenesis of SSc. However, this should be confirmed in a larger number of patients and with validated methods capable of specifically measuring products of complement activation.

We recently established a novel enzyme-linked immunosorbent assay (ELISA) that can reliably detect soluble C4d in plasma [12]. In the present study, we re-examined the usefulness of C4d as biomarker in a cohort of 122 patients with SSc. Also, we measured a soluble marker of alternative pathway activation, C3bBbP, and soluble TCC (sTCC), a marker of the final step of complement activation via any pathway in the same cohort. In order to compare the results obtained for patients with SSc with those for patients with other autoimmune diseases in whom activation of the complement system was previously reported, we performed the same analyses in patients with rheumatoid arthritis (RA) [13], psoriatic arthritis (PsoA) [14] and ankylosing spondylitis (AS) [15], as well as in healthy control subjects.

## Methods

### Patients

Plasma samples were collected from 122 patients who fulfilled the American College of Rheumatology (ACR) criteria for SSc [16]. The disease was classified as dcSSc (*n* = 41) or lcSSc (*n* = 81), based on the extent of skin involvement [3]. Samples were collected within a mean (±SD) of 7.5 (8.5) years of disease onset, which was defined as the first non-Raynaud’s manifestation. A summary of baseline characteristics of patients with SSc is presented in Table 1. Healthy control plasma samples were collected from 47 age-matched (mean 52.3 ± 8.7 years) and sex-matched (14 males, 33 females) healthy volunteers with no history of rheumatologic disease. All plasma samples were retrieved in a standardised fashion (non-fasting) and were stored at −80 °C after centrifugation until the experiments. Additionally, we included plasma samples from age- and sex-matched patients with RA (*n* = 81), PsoA (*n* = 22) and AS (*n* = 20). All patients with RA fulfilled the 1987 ACR criteria [17]. Diagnoses of PsoA and AS were based on clinical judgment by the treating physician and included radiographic examinations when applicable. All patients with AS had axial involvement but no clinical signs of peripheral arthritis, whereas the patients with PsoA had peripheral arthritis but no clinical signs of axial involvement.

**Table 1:** Clinical features at the time of blood sampling of 122 patients with systemic sclerosis

Feature	Data
Age, years	56.4 (14.8)
Disease duration, years	7.5 (8.5)
mRSS, points	11.1 (11.0)
Sex, male/female, *n* (%)	22 (18)/100 (82)
Subset, lcSSc/dcSSc, *n* (%)	81 (66)/41 (34)
Ab, ACA/ATA/ARA/ANA^+^/ANA^−^, *n* (%)	34 (28)/22 (18)/17 (14)/40 (33)/9 (7)

*Abbreviations*: *Ab* Autoantibodies, *mRSS* Modified Rodnan skin score, *lcSSc* Limited cutaneous systemic sclerosis, *dcSSc* Diffuse cutaneous systemic sclerosis, *Ab* Autoantibodies, *ACA* Anti-centromere, *ATA* Anti-topoisomerase I, *ARA* Anti-RNA polymerase III, *ANA* ^*+*^ Anti-nuclear but not anti-centromere, anti-topoisomerase I or anti-RNA polymerase III autoantibodies, *ANA* ^*−*^ No anti-nuclear autoantibodies

### Clinical data

Anti-nuclear antibodies (ANA) and anti-centromeric antibodies (ACA) were analysed by an indirect immunofluorescence technique using the human Hep-2 cell line as a substrate; anti-topoisomerase I antibodies (ATA) were analysed by ELISA; and anti-RNA polymerase III antibodies (ARA) were analysed by immunoprecipitation. Serum cartilage oligomeric matrix protein (COMP) was measured with a commercial sandwich ELISA using two monoclonal antibodies directed against separate antigenic determinants (AnaMar, Lund, Sweden) [18]. Subjects were classified as having dcSSc or lcSSc according to LeRoy et al.’s criteria [3]. The severity of skin involvement was determined by the modified Rodnan skin score (mRSS) [19]. Disease duration was determined as the time from the first non-Raynaud’s manifestation.

### Measurement of complement activation markers

Plasma concentrations of C4d [12], C3bBbP [20] and sTCC [20] were measured by specific sandwich ELISAs in plasma ethylenediaminetetraacetic acid (EDTA) samples as described previously. Readout of each of these assays was given in complement activation units (CAU), a defined arbitrary unit set for the International Complement Standard #2 (ICS#2) sample, which is serum-pooled from about 1000 healthy individuals and incubated with activators of all three complement pathways [20].

### Haemolytic assays

Plasma EDTA samples collected from patients with SSc were tested for activity of classical or alternative complement pathways by haemolytic assays performed as described elsewhere [21], with small modifications. Briefly, the classical complement pathway was activated on antibody-sensitised sheep erythrocytes. To overcome the inhibitory effect of EDTA, plasma samples were diluted 1:100 in DGVB^2+^ buffer (2.5 mM veronal buffer, pH 7.35, 72 mM NaCl, 140 mM glucose, 0.1% gelatin, 1 mM MgCl_2_ and 0.15 mM CaCl_2_). For the alternative pathway, plasma samples were diluted 1:20 in Mg-ethylene glycol-bis(2-aminoethylether)-*N*,*N*,*N′*,*N′*-tetraacetic acid (Mg-EGTA) buffer (2.5 mM veronal buffer, pH 7.3, 70 mM NaCl, 140 mM glucose, 0.1% gelatin, 7 mM MgCl_2_ and 10 mM EGTA) and added directly to rabbit erythrocytes.

### Immunohistochemical staining of C3

Kidney and skin biopsies collected from patients with SSc were fixed in formalin and embedded in paraffin. Sections of 4-μm thickness were cut and automatically pre-treated using the PT Link system (Dako/Agilent Technologies, Santa Clara, CA, USA) and then stained in an Autostainer Plus (Dako) with rabbit anti-human C3c polyclonal antibody (P0062; Dako) at a final dilution of 1:5000 for 30 minutes. Subsequently, the EnVision Flex HRP kit (Dako) was applied to the sections for 20 minutes to detect primary antibodies, followed by 3,3′-diaminobenzidine (DAB) reagent for visualisation.

### Statistical analysis

Obtained data on the appearance of complement activation markers and haemolytic activity were not distributed normally; in each complement measurement there were outliers, and some patient subgroups were rather small. Therefore, we used non-parametric statistical methods throughout the whole analysis. The Kruskal-Wallis and Mann-Whitney *U* tests were used to compare multiple groups or two groups, respectively. Spearman’s correlation was used to analyse relationships. *p* values <0.05 were considered significant. Calculations were performed with Prism 5.0 (GraphPad Software, La Jolla, CA, USA) and IBM SPSS Statistics version 20 (IBM, Armonk, NY, USA) software.

## Results

### The complement system is activated in patients with autoimmune diseases

**Fig. 1:**
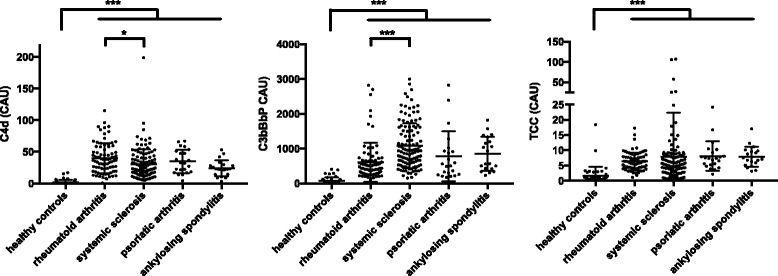
Complement activation markers in systemic sclerosis and other autoimmune diseases. Graphs show mean and standard deviation of C4d (*left panel*), C3bBbP (*middle panel*) and soluble terminal complement complex (TCC) (*right panel*) concentrations. Value obtained for each patient (depicted with single points) is an average of two independent experiments. Statistical analysis was performed by Kruskal-Wallis test with Dunn’s multiple comparisons post-test, and statistical significance was set at *p < 0.05 and ***p < 0.001.Abbreviation: *CAU* Complement activation units

We tested three different complement activation markers: (1) C4d, corresponding to activation of the classical pathway; (2) the fluid-phase alternative C3 convertase (C3bBbP), reflecting activation of the alternative pathway as well as the amplification loop enhancing the cascade at the level of convertases; and (3) sTCC, which evaluates the lytic (terminal) pathway (Fig. 1). All autoimmune patients, regardless of diagnosis, had 5- to 15-fold increased levels of all three markers, thus confirming ongoing complement activation. Regarding SSc, C4d was increased to a similar degree as for AS and PsoA, but to a lesser extent than in RA (*p* = 0.05 by Kruskal-Wallis test). Interestingly, the alternative pathway was activated significantly more strongly in patients with SSc than in patients with RA (*p* < 0.001). All groups of patients showed similar levels of sTCC. As expected, healthy control subjects showed low levels of all three markers.

### Correlations of complement activation markers in patients with autoimmune diseases

Because sTCC is a marker of the terminal complement pathway, which is fuelled by all complement pathways, we analysed which of the early complement markers correlate to appearance of sTCC in patients with SSc and other autoimmune diseases. Only C3bBbP, and not C4d, was significantly correlated to sTCC in all groups of patients. However, in patients with RA, C4d was significantly correlated to C3bBbP, which may indicate the role of the amplification of the classical pathway via alternative convertases in overall complement activation in this patient group. Correlation coefficients and *p* values are given in Table 2.

**Table 2:** Correlations between complement biomarkers C4d, C3bBbP and terminal complement complex in plasma samples from patients with autoimmune diseases

Disease	C4d-TCC correlation		C3bBbP-TCC correlation		C4d-C3bBbP correlation	
	Spearman’s *r*	*p* value	Spearman’s *r*	*p* value	Spearman’s *r*	*p* value
SSc (*n* = 122)	−0.06	0.47	0.47	<0.0001^a^	0.099	0.27
RA (*n* = 81)	0.12	0.29	0.41	0.0001^a^	0.26	0.018^b^
PsoA (*n* = 22)	0.035	0.88	0.52	0.013^b^	0.086	0.70
AS (*n* = 20)	−0.33	0.15	0.52	0.019^b^	−0.20	0.40

*Abbreviations: AS* Ankylosing spondylitis, *PsoA* Psoriatic arthritis, *RA* Rheumatoid arthritis, *SSc* Systemic sclerosis, *TCC* Terminal complement complex

^a^ *p* <0.001

^b^ *p* < 0.05

### Correlations of complement activation markers to mRSS and COMP

To find out whether complement activation correlates to the severity of fibrosis expressed by mRSS, we performed Spearman’s correlation analysis and found weak, borderline significant correlation between mRSS and C4d (Table 3). Furthermore, COMP, which is expressed by fibroblasts in SSc, is associated with mRSS [22] (in the present cohort, Spearman’s *r* = 0.47, *p* = 0.0005) and is known to activate the alternative pathway [23]. However, we did not observe a statistically significant correlation between complement activation markers and COMP levels in plasma, implying that it is likely not a major complement trigger in these patients, at least not when released into blood.

**Table 3:** Correlations of complement activation markers with age, modified Rodnan skin score and cartilage oligomeric matrix protein

	*n*	C4d	*p* value	C3bBbP	*p* value	TCC	*p* value
Age	122	−0.14	0.13	−0.083	0.36	0.017	0.86
mRSS	118	0.17	0.06	−0.16	0.087	−0.082	0.38
COMP	53	0.16	0.26	−0.052	0.71	0.092	0.51

*Abbreviations: COMP* Cartilage oligomeric matrix protein, *mRSS* Modified Rodnan skin score, *TCC* Terminal complement complex

### Analyses of complement activation markers in the subgroups of patients with SSc

We classified the patients into subgroups depending on the form of disease (lcSSc vs. dcSSc); typical complications, such as pulmonary arterial hypertension (PAH), scleroderma renal crisis (SRC), pitting scars, ulcers, telangiectasias; or the presence of certain types of anti-nuclear autoantibodies, including ACA, ATA, ARA, ANA without ACA, ATA or ARA, and finally ANA-negative. We did not observe significant differences in C4d, C3bBbP and sTCC levels between men and women or between patients with lcSSc and those with dcSSc (Table 4). Also, concentrations of these markers were equally distributed within patients with and without PAH, pitting scars, ulcers, and telangiectasias. Importantly, a different distribution of complement activation markers was detected in patients with SRC, who had significantly higher amounts of C4d (*p* = 0.036) and significantly lower levels of C3bBbP (*p* < 0.001) and sTCC (*p* = 0.003) than patients without kidney involvement. When overall distribution of autoantibodies was analysed by the Kruskal-Wallis test, we found a significant difference for sTCC (*p* = 0.016) and a difference on the border of significance for C3bBbP (*p* = 0.055). Although not statistically significant (*p* = 0.095), there was a trend for C4d, according to which patients without any antinuclear autoantibodies presented with low levels of this marker. Furthermore, patients with ARA positivity showed a pattern of complement activation markers similar to that of the patients with SRC (high C4d, low C3bBbP and TCC). Additional Kruskal-Wallis analysis performed for a single type of autoantibody revealed that the ARA-positive group differed significantly from patients without any antinuclear antibodies (ANA-negative) at a *p* level <0.05 for all complement markers. Also, the ARA-positive group had significantly lower amounts of C3bBbP and TCC than ARA-negative patients (*p* < 0.01). Interestingly, 8 (47%) of 17 patients with ARA presented also with SRC, a group that accounted only for 15% of all patients with SSc (18 of 122). Taken together, patients with ARA were, as expected, overrepresented among those with SRC and, importantly, manifested the same pattern of complement activation markers.

**Table 4:** Analyses of complement activation markers in the subgroups of patients with systemic sclerosis

	*n*	C4d, mean (SD)	*p* value	C3bBbP, mean (SD)	*p* value	TCC, mean (SD)	*p* value
Sex			0.96		0.20		0.87
Male	22	35.6 (40.3)		913 (574)		5.80 (3.69)	
Female	100	29.2 (18.3)		1119 (661)		8.39 (15.8)	
Subset			0.88		0.11		0.36
lcSSc	81	30.1 (25.2)		1143 (655)		8.56 (16.4)	
dcSSc	41	30.8 (20.7)		962 (629)		6.66 (9.34)	
PAH			0.19		0.75		0.76
No	96	31.8 (25.3)		1069 (645)		7.50 (12.4)	
Yes	26	25.0 (16.0)		1131 (676)		9.49 (20.5)	
SRC			0.036		<0.001		0.003
No	104	28.9 (23.7)		1156 (655)		8.57 (15.4)	
Yes	18	38.6 (22.9)		656 (423)		4.22 (5.54)	
Ab			0.095		0.055		0.016
ACA	34	30.7 (16.9)		1029 (594)		5.83 (5.70)	
ATA	22	32.7 (18.8)		1140 (744)		10.8 (22.0)	
ARA	17	35.0 (22.5)		761 (560)		3.19 (2.59)	
ANA^+^	40	29.5 (32.2)		1195 (642)		10.6 (18.0)	
ANA^−^	9	18.1 (10.0)		1244 (705)		5.62 (3.36)	
Pitting scar			0.50		0.35		0.76
No	68	31.0 (26.1)		1071 (658)		9.30 (18.6)	
Yes	50	28.6 (19.0)		1155 (633)		6.53 (5.62)	
Ulcer			0.35		0.36		0.54
No	105	30.5 (23.9)		1090 (656)		8.31 (15.3)	
Yes	14	25.5 (17.8)		1183 (574)		6.19 (6.53)	
Telangiectasias			0.83		0.19		0.39
No	56	28.5 (17.8)		1003 (586)		6.07 (3.80)	
Yes	63	31.2 (27.2)		1189 (687)		9.84 (19.6)	

*Abbreviations*: *PAH* Pulmonary arterial hypertension, *SRC* Scleroderma renal crisis, *Ab* Autoantibodies, *ACA* Anti-centromere, *ATA* Anti-topoisomerase I, *ARA* Anti-RNA polymerase III, *ANA* ^*+*^ Anti-nuclear but not anti-centromere, anti-topoisomerase I or anti-RNA polymerase III, *ANA* ^*−*^ No anti-nuclear autoantibodies, *dcSSc* Diffuse cutaneous systemic sclerosis, *lcSSc* Limited cutaneous systemic sclerosis, *TCC* Terminal complement complex

Autoantibody analyses were by Kruskal-Wallis test; all others were by Mann-Whitney *U* test

### Haemolytic activity of plasma samples collected from patients with SSc

**Fig. 2:**
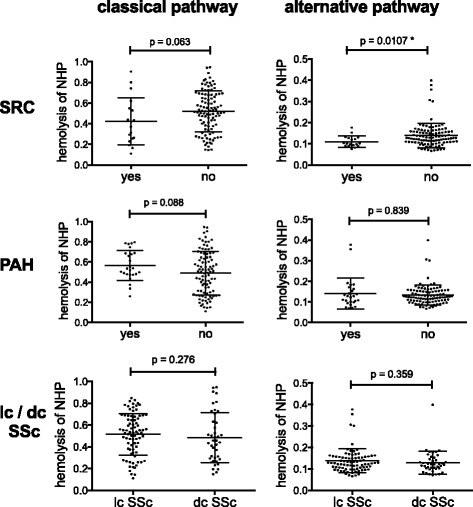
Haemolytic activity of plasma samples collected from patients with systemic sclerosis. *Left column* shows classical pathway haemolytic activity of 1% plasma, and *right column* shows alternative pathway activity of 5% plasma. Value of 1 on *y*-axis corresponds to the readout of normal human plasma (NHP). Results are means ± standard deviation derived from two independent experiments, and statistical significance is set at *p < 0.05 according to Mann-Whitney *U* test.Abbreviations: *SRC* Scleroderma renal crisis, *PAH* Pulmonary arterial hypertension, *lcSSc* Limited cutaneous systemic sclerosis, *dcSSc* Diffuse cutaneous systemic sclerosis.  Graphs comparing the patients with vs. without ulcers, pitting scars and telangiectasias are not shown, because the differences between groups did not reach statistical significance

The levels of C3bBbP and sTCC in the SRC group (means 656 CAU and 4.22 CAU, respectively) were lower than in other patients with SSc; however, these levels were still considerably higher than analogous means for healthy control subjects (79.7 CAU and 1.64 CAU, respectively), indicating that complement activation takes place during the renal crisis. At the same time, C4d, which is a marker of classical pathway activation and the end-degradation product of C4b component, increased to significantly higher levels than in the non-SRC group. One possible explanation for these observations is that during SRC the classical pathway is strongly initiated by autoantibodies, and then an amplification loop driven by the alternative pathway takes over the cascade, leading to systemic depletion of its components, including C3 and factor B proteins. Such depletion is also reflected in a reduction of soluble sTCC compared with patients with no SRC episodes. To confirm the scenario of ongoing complement consumption in SRC, we analysed haemolytic activity of plasma samples from patients with SSc. Residual complement activity was examined in assays testing the classical and alternative pathways (Fig. 2). Of all classifications made for the whole SSc cohort, only the SRC-positive group showed significantly lower haemolytic activity in the alternative pathway (*p* = 0.0107) and a borderline significant drop of haemolytic activity in the classical pathway (*p* = 0.063), which confirmed systemic complement depletion, most probably at the level of alternative convertases.

### C3 staining in kidney biopsies from patients with SRC

**Fig. 3:**
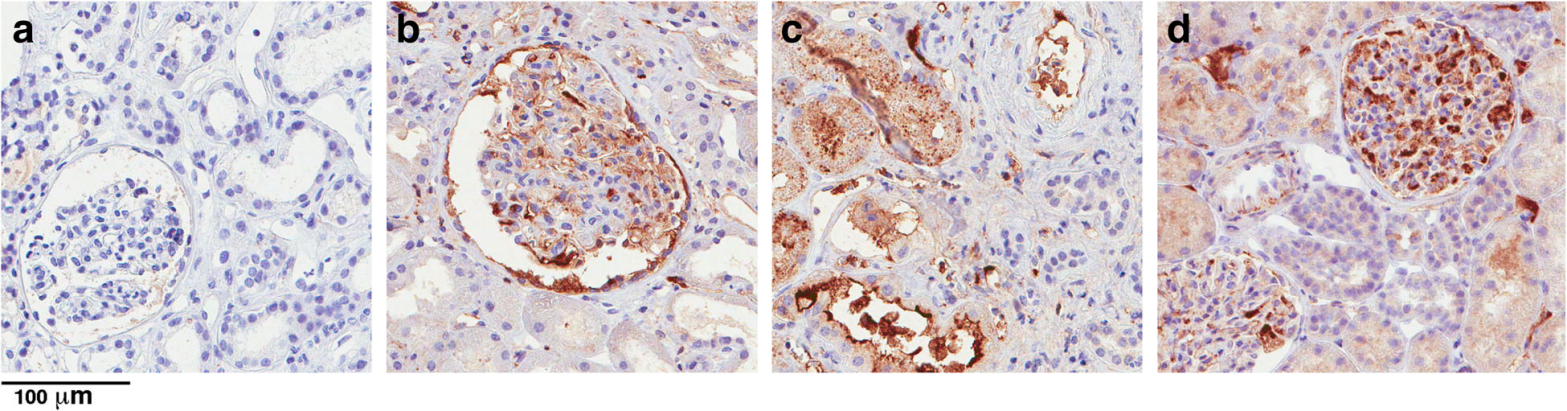
C3b deposition in kidney biopsies from patients with scleroderma renal crisis (SRC). Shown are representative micrographs of kidney biopsy cores immunostained for C3b. **a** An example of a biopsy obtained from a patient with SRC, which was negative for C3b. **b** Example of a biopsy positive for C3b in glomeruli. **c** Example of a biopsy positive for C3b in tubules. **d** Positive control biopsy from patient diagnosed for immunoglobulin A nephropathy with distinct glomerular C3 positivity

Because the SRC subgroup showed a distinct pattern of complement activation markers, we searched for the primary source of a putative strong complement activation deduced from lower levels of C3bBbP and lower haemolytic activity of plasma samples. Kidney biopsies from 5 of 18 patients with SRC included in the study were available, and we examined these for C3b deposition by immunohistochemistry. C3b deposits were detected in glomeruli of two patients and in tubules of one more patient (Fig. 3), whereas C3b staining was negative for kidneys of two remaining patients. These findings indicate that ongoing complement activation in kidneys may be one of the hallmarks of SRC. However, it does not explain all cases, and other sources of activation are also plausible.

### C3b staining in skin biopsies from ARA^+^, ARA^−^ and ARA^−^/ACA^+^ patients

**Fig. 4:**
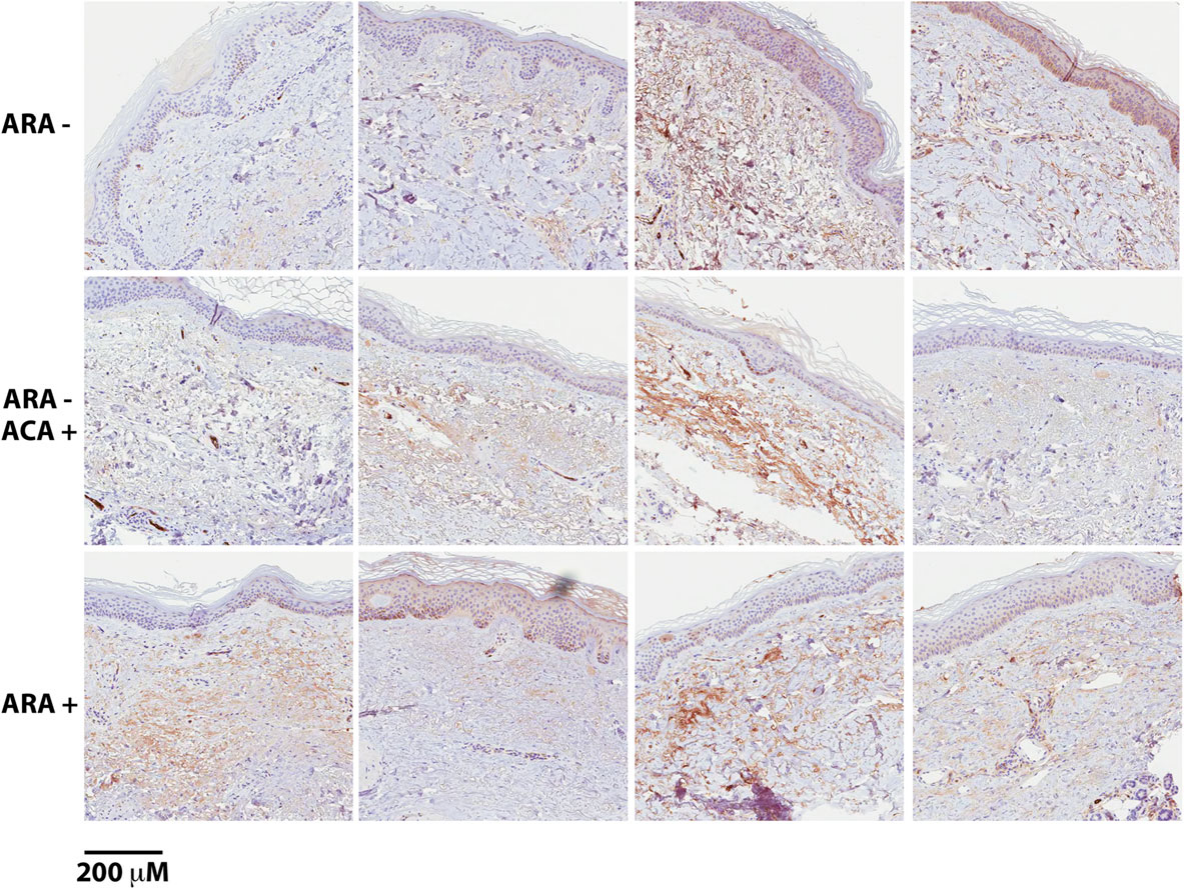
C3b staining of skin biopsied from anti-RNA polymerase III (ARA^+^), ARA^−^ and anti-centromere-positive (ACA^+^)/ARA^−^ patients. Shown are representative micrographs of skin biopsies immunostained for C3b. Four patients per group (rows) were available for the study. In most images, the endothelium of the dermal arterioles is distinctly positive. There is also a fainter staining associated with the dermal collagen strands. In the basal layer of the epidermis, melanocytes are seen, filled with melanin pigment but negative for C3b

Searching for further explanations for complement activation in patients with SRC, we followed the overrepresentation of ARA-positive patients in the SRC group and analysed C3b deposition in skin biopsies obtained from ARA^+^, ARA^−^ and ARA^−^/ACA^+^ patients. Material from four patients in each group was available. We observed C3b deposition in skin tissue from all ARA^+^ patients, but other groups also had at least two of four slides that were positive (Fig. 4), so there was no dramatic difference between the groups. The staining was localised to the dermal layer and most prominent in the endothelium of small dermal arteries. Less staining was associated with the collagenous fibres of the dermis. In the basal layer of the epidermis, cells were discovered containing what seemed to be stain. However, these cells were melanocytes, and the pigment was melanin, not DAB chromogen (Fig. 4).

## Discussion

Reliable measurement of complement activation is not a trivial task. Of two possible strategies, measurement of native complement protein consumption and measurement of markers appearing only after complement activation, the latter seems to be more reliable. This is due partly to large interpersonal differences in some complement proteins, such as that normal concentration of the main complement protein C3 ranges from 0.8 to 1.8 mg/ml. On one hand, these levels are affected by both consumption and a changing rate of synthesis because many complement factors are acute-phase response proteins. On the other hand, challenges in the strategy of measuring products of activation relate to the specificity of antibodies, which must target only neoepitopes localised on processed complement proteins and not epitopes on ubiquitous, native molecules. Previous studies performed with 34 patients with SSc revealed higher levels of C4d in blood of patients with dcSSc than in patients with lcSSc, suggesting that C4d content may be associated with clinical severity of SSc [10]. However, the authors of that study did not use specific anti-C4d antibodies; instead, they performed precipitation of high-molecular-weight plasma proteins and detected C4d in supernatant with whole antiserum by nephelometry. We re-examined this hypothesis using plasma samples of 122 patients with our recently designed C4d ELISA. The advantage of this method over nephelometry and other, previously used C4d immunoassays is that it uses a novel antibody specific to a 5-aa linear cleavage site neoepitope. Previously, we showed that the readout of this assay is unaffected by repeated freezing-thawing or heating of samples, which is not the case for some other techniques based on conformational neoepitopes [12]. We noticed a clear increase of C4d as well as the complement activation markers C3bBbP and sTCC in patients with SSc as well as in patients with other rheumatic diseases compared with healthy control subjects (Fig. 1). However, we could confirm neither a link between clinical severity of SSc and C4d content nor associations of complement activation markers with particular syndromes characteristic for SSc, except for SRC.

Renal crisis is reported typically in 5–10% patents with SSc [24, 25]. Historically, this group of patients had a high risk of death (up to 85% mortality after 1 year [26]), and even after introducing treatment with angiotensin-converting enzyme inhibitors, mortality remains relatively high at 18% after the first year and 41–58% after 5 years [27, 28]. To prevent loss of kidney function and other associated complications, such as systemic hypertension, retinopathy or pulmonary oedema, patients require early identification and aggressive treatment [25]. Therefore, increasing knowledge about underlying pathomechanisms of SRC is necessary. Our data suggest that activation of complement, which is a feature common to rheumatic and/or autoimmune diseases [13, 29], presents differently in patients with SSc with SRC. Another important observation was that the same pattern of complement activation markers seen in SRC was observed in the group of ARA-positive patients, which accounted for 44% of patients with SRC in our study and 33% [30] to 59% [27] in other studies. Trends and a significant increase of C4d marker in these two groups of patients linked to each other speak for enhanced activation of the classical pathway. C3bBbP is a marker that may indicate activation of the alternative pathway alone or as an amplification loop of the classical or lectin pathway. At first glance, elevated C4d and decreased C3bBbP levels in the same patients may appear contradictory, but the nature of these markers may provide a logical explanation. Whereas C4d is the end degradation product of an early component of the classical pathway C4b and may accumulate over time, C3bBbP is a fluid-phase convertase, an enzymatic complex that converts available C3 into C3b. Activation of the alternative pathway leads to depletion of C3 and factor B and, by doing so, limits de novo formation of C3bBbP. However, C3bBbP decays both spontaneously and with the aid of several complement inhibitors [20]. As a result of limited replacement of alternative pathway components and pathway exhaustion, patients with acute episodes of complement activation may present with lower C3bBbP content than those with chronic disease. The same is true for sTCC.

On the basis of our results, we hypothesised that acute complement activation takes place during the onset of SRC and that depletion of available complement is another consequence of such scenario. Our hypothesis was confirmed by haemolytic assays in which residual complement activity of plasma from patients with SRC was significantly lower than that in non-SRC subjects. In conjunction with a previous report showing increased C4d deposition in peritubular capillaries of patients with SSc with SRC compared with normotensive controls and hypertensive non-SRC control subjects [24], we should consider local complement activation as an important element of the SRC pathomechanism. This hypothesis is strengthened by presence of C3b deposits, which reflect complement activation via all pathways, in three of five analysed kidney biopsies from patients with SRC. We also tested whether ARA-positive patients overrepresented in the SRC group had increased C3b deposits in skin. Although all ARA-positive individuals showed C3b staining in skin biopsies, we also found it in some ARA-negative patients. Therefore, we hypothesise that local complement activation in kidneys and skin takes place during SRC, but that this phenomenon is probably not limited only to this subgroup and that other, distinct features may be characteristic for renal involvement. Nonetheless, complement activation may be a strongly contributing factor in some patients with SRC, and these patients could be considered for treatment with emerging complement inhibitors such as eculizumab, which is now used in therapy for another kidney disease with complement involvement: haemolytic uremic syndrome.

## Conclusions

The present study shows that biomarkers of activation of all complement pathways measured with improved assays were increased in patients with SSc compared with healthy control subjects. Patients with SSc with SRC showed a distinct pattern of complement markers indicating ongoing complement consumption. This, finding, together with glomerular deposits of C3b found in some of these patients, indicates that complement activation is an important feature of SRC.

## Abbreviations

ACA: Anti-centromere
ACR: American College of Rheumatology
ANA: Anti-nuclear
ARA: Anti-RNA polymerase III
AS: Ankylosing spondylitis
ATA: Anti-topoisomerase I
CAU: Complement activation units
COMP: Cartilage oligomeric matrix protein
DAB: 3,3′-Diaminobenzidine
dcSSc: Diffuse cutaneous systemic sclerosis
EDTA: Ethylenediaminetetraacetic acid
EGTA: Ethylene glycol-bis(2-aminoethylether)-*N*,*N*,*N′*,*N′*-tetraacetic acid
ELISA: Enzyme-linked immunosorbent assay
lcSSc: Limited cutaneous systemic sclerosis
mRSS: Modified Rodnan skin score
NHP: Normal human plasma
PAH: Pulmonary arterial hypertension
PsoA: Psoriatic arthritis
RA: Rheumatoid arthritis
SRC: Scleroderma renal crisis
SSc: Systemic sclerosis
sTCC: Soluble terminal complement complex
TCC: Terminal complement complex
